# Failure Analysis of TEVG’s II: Late Failure and Entering the Regeneration Pathway

**DOI:** 10.3390/cells11060939

**Published:** 2022-03-10

**Authors:** Maria A. Rodriguez-Soto, Alejandra Riveros, Natalia Suarez Vargas, Andres J. Garcia-Brand, Carolina Muñoz Camargo, Juan C. Cruz, Nestor Sandoval, Juan C. Briceño

**Affiliations:** 1 Department of Biomedical Engineering, Universidad de los Andes, Bogotá 111711, Colombia; ra.riveros11@uniandes.edu.co (A.R.); na.suarez122@uniandes.edu.co (N.S.V.); aj.garcia14@uniandes.edu.co (A.J.G.-B.); c.munoz2016@uniandes.edu.co (C.M.C.); jc.cruz@uniandes.edu.co (J.C.C.); 2 Department of Congenital Heart Disease and Cardiovascular Surgery, Fundación Cardio Infantil Instituto de Cardiología, Bogotá 111711, Colombia; nsandoval@cardioinfantil.org; 3 Department of Research, Fundación Cardio Infantil Instituto de Cardiología, Bogotá 111711, Colombia

**Keywords:** tissue-engineered vascular grafts, regeneration, late failure, immune response, atherogenesis, intimal hyperplasia

## Abstract

Tissue-engineered vascular grafts (TEVGs) are a promising alternative to treat vascular disease under complex hemodynamic conditions. However, despite efforts from the tissue engineering and regenerative medicine fields, the interactions between the material and the biological and hemodynamic environment are still to be understood, and optimization of the rational design of vascular grafts is an open challenge. This is of special importance as TEVGs not only have to overcome the surgical requirements upon implantation, they also need to withhold the inflammatory response and sustain remodeling of the tissue. This work aims to analyze and evaluate the bio-molecular interactions and hemodynamic phenomena between blood components, cells and materials that have been reported to be related to the failure of the TEVGs during the regeneration process once the initial stages of preimplantation have been resolved, in order to tailor and refine the needed criteria for the optimal design of TEVGs.

## 1. Introduction

Cardiovascular disease (CVD) comprises a series of disorders that affect the heart and the vascular system, causing an estimated 17.9 million deaths worldwide every year [1]. The pathophysiology of vascular diseases is characterized by either the blockage of blood vessels or by degenerative processes in the vascular wall that elicit detrimental changes in their elasticity that ultimately compromise the blood flow [2]. As patients with CVDs may require surgical interventions to recover or redirect the blood flow, the replacement of the diseased vessel with vascular grafts (VGs) remains the primary alternative. The preferred choice for VG used in the replacement of small vessels (<6 mm) is autologous vessels such as the saphenous vein (SV) and internal thoracic artery (ITA). Regardless of the benefits imparted by their implantation, critical issues of availability and morbidity limit their implementation as suitable as VGs [3,4]; thus, commercially available synthetic vascular grafts provide a less than ideal alternative related to the lack of the needed cues for regeneration.

The most popular synthetic VGs in the market are made from polytetrafluoroethylene (PTFE—also known as Teflon^®^) and polyethylene terephthalate (PET—also known as Dacron^®^). However, despite being accessible and useful, their long-term success is limited, as it can be seen by the decreased patency rates below 32% after 2 years in small-diameter vessels or complex hemodynamic conditions [5,6], which are closely related with the need of reintervention. Thus, to provide an appropriate treatment being able to reduce future reinterventions, there is an open challenge to design an alternative to the use of synthetic vascular grafts, whose design should meet the desired criteria to impart an appropriate treatment by using regenerative medicine and tissue engineering as useful tools to develop novel approaches called tissue-engineered vascular grafts (TEVGs). These strategies have tailored the blood vessel architecture and functionalities aiming to restore the operative conditions and structure of the diseased vessel. Nevertheless, to our knowledge, only Artegraft is a successful example of a rationally designed graft based on decellularized bovine carotid artery [7], which elucidates the need for a further understanding of the failure causes founded in the preclinical testing of TEVGs to rationally guide the desired response.

Although the pathophysiology of the autologous and synthetic VG and some TEVGs has been reported, the phenomena leading one to overcome failure remains partially unclear, leading to a limited translation of TEVGs to clinical trials [8]. In fact, TEVGs have encountered translation obstacles at preclinical models where patency loss is attributed to the appearance of development cell phenotypes in the vascular wall that interfered with the proper regeneration signals required for an accurate compliance of the vascular wall under hemodynamic conditions [9,10]. In addition, the failure of TEVGs has a multifactorial origin, so in most cases the preclinical studies do not generally evaluate given the complexity of the regenerative profile, which indicates the limited information of the effect of the implanted TEVGs with the microenvironment. For that reason, the comprise of the cumulative impact of the blood–material–cells interaction seems to have a great impact on the design of future TEVGs with appropriate modulation attributes [11].

Considering that cell–material interactions in TEVGs depend mainly on the biochemical and mechanical stimuli under hemodynamic conditions, each cell phenotype present in the vascular wall, the peripheral tissues and the blood should differentially activate signaling pathways according to the stimuli signals and their intensity, especially in those related to the immuno-regenerative crosstalk [12,13]. From acute inflammation to the remodeling of the vascular wall, a series of events occur upon implantation in which the TEVGs should meet the specific biocompatibility criteria to overcome possible failure causes to find the balance in the relationships of those events to fulfill regeneration. Moreover, as cell behavior and crosstalk are affected by molecular interactions such as chemosignaling, there are few studies that report that the molecular interactome profile characterized through transcriptomics, proteomics and metabolomics could help to a better definition of the targets in TEVGs regeneration. However, the completely set represents a gap in the literature regarding the key issues that must be considered for the TEVGs’ success [8].

Therefore, as an attempt to establish a guideline of the events that follow the implantation of the TEVGs, and provide a baseline of the biomolecular events occurring through the regeneration pathways in either ideal successful pathways or failure processes, here we aim to highlight several considerations based on preclinical stages of TEVGs studies in order to guide future translational studies by including a comprehensive evaluation of the integration progress of the TEVG through trending molecular tools [14].

## 2. Materials and Methods

In an effort to provide an organized logical sequence of events within the integration process of a TEVG, our research group proposes a series of phases related to the commonly evaluated timepoints of preclinical studies. In this sense, Phase I is the peri-implantation period in which the success is defined by the graft overcoming the surgery and postoperative care. Phase II is the period after implantation with acute inflammation in which success is defined by an initial induction of endothelialization and a final transition to a regenerative environment. Finally, Phase III is the vascular wall remodeling and device maturation in which success is defined by the graft as an optimum substrate for cells in the vascular wall developing functional phenotypes in the context of a compliant vessel. Accordingly, for each of these phases, we grouped different metabolic pathways that could result in failure and named these groups as cases within the phases. In this way, we aimed to characterize each of the metabolic pathways in terms of the biomolecular interactions of the TEVG with the implant microenvironment and establish the key markers that need to be evaluated in translational studies.

To determine the specific bio-molecular events occurring during these phases, we performed a literature review, and a search of documents was carried out in PubMED, Access Medicine, Annual Reviews, EBSCO, EMBASE, *Nature*, Science Direct, SCOPUS, Springer, Springerlink, and Wiley Online Library. The used keywords were “vascular grafts”, “Pathophysiology”, “cell behavior”, “blood components”, “biomimetic”, “Biomaterials”, “failure”, “Design “, “approaches”, “wall shear stress” and “gene expression”. The Boolean operator AND was the connector for the search. Only documents published in English were considered. Only articles in which the main body contents and/or abstract accounted for detailed cell behavior and blood-extracellular matrix under physiological and pathological conditions induced by the TEVG material and/or hemodynamics were selected. The collected documents were sorted out according to the relevance of the development and stage of testing and 101 documents were selected. Then, information was further organized by looking at the interactions of between pairs of factors and their direct impact on the phases of TEVG integration and remodeling.

## 3. Early and Late Interaction of TEVGs with the Biological Environment

The objective of tissue-engineered vascular grafts is to remain effective in sustaining perfusion in the long term; however, both regenerative and non-regenerative vascular grafts have to overcome the inflammatory conditions of the peri implantation period. These interactions fall within Phase I of our designed timeline. The first interaction of the TEVG with the native tissues and blood is related to the homeostasis maintenance. When the TEVG lacks the required biocompatibility, thrombogenesis and an exacerbated acute inflammatory response are the first causes of TEVG failure [11,15].

When aiming to assess the capacity of the graft to modulate the integration capacity of the graft to the surrounding tissue and remain patent through the process, evaluating TEVGs within Phase II and III should consider the specific metabolic pathways activated by the interactions between the graft and the microenvironment.

After the first months to the first two years after implantation, the main responses are related to the resolution of the inflammatory process and tissue ingrowth. Furthermore, failure causes are associated with the absence of homeostatic remodeling of the vascular wall. Consequently, non-physiological compensation mechanisms to try to maintain perfusion activate. These mechanisms could lead to changes in the mechanical properties of the graft and in some cases to patency loss. Depending on the nature of the misbalance of tissue remodeling, patency loss can be related to intimal hyperplasia (IH), in which smooth muscular cells overproliferate [16,17], or atherosclerosis, in which calcification occurs [18,19], both leading to a decrease in the TEVG lumen. Whether or not there are surgical interventions to recover the proper blood flow, a non-biocompatible TEVG will fail over time.

## 4. Integration of a Vascular Grafts In-Vivo: Challenges and Opportunities

Aiming to recognize the challenges and opportunities, to establish a baseline and a series of indicators that translational studies should consider for the design and evaluation of TEVGs in each of the three phases defined previously for pre-clinical models in light of the TEVG triad [20], here we provide a complete description of the critical targets in regeneration that might promote regeneration or unchain events related to the patency loss or failure of vascular grafts. To this end, here we report different alternative cases that recapitulate the outcomes of favorable or non-favorable interactions. In summary, we provide the description of a case in which the biomolecular events result in a non-regenerative response and/or patency loss due to the incompatibility of the TEVG, and a case that describes the ideal conditions in which the TEVG biocompatibility provides the biochemical and mechanical stimuli required to overcome the phase and move towards the regenerative pathway. Furthermore, here we show intermediate situations evidenced by patency loss and other clinical signs and symptoms related to TEVG failure. This mechanistic explanation of the interaction of the TEVGs with the native environment might be used as a tool to recognize reference events for the rational design of TEVGs.

Failure after the peri-implantation period on phases II and II has been attributed to the persistence of chronic inflammation. Here, we start from the concept that a TEVG can only initiate a regenerative pathway once the inflammatory response has been stabilized and the microenvironment conditions around the graft are suitable to start tissue remodeling. After this point, the TEVG will begin a repopulation process that, if balanced, could end up in the integration of the TEVG to the native vascular tree. Finally, after overcoming phase II, possible causes of failure are related to altered hemostasis of cell proliferation and non-desirable phenotype expression, whereas success appears to be related to the ability of the TEVG to allow cell migration and induce the remodeling processes of the vascular wall.

## 5. Phase II: Period after Implantation—First Months

For the TEVG to reach Phase II, the first immune responses should have stabilized with absence of observable occlusion. According to the biomaterial properties, the microstructure and the hemodynamic behavior within the TEVG, the developed pathway is dependent on the recovery of the homeostatic balance in the system to avoid thrombogenesis and induce the immune response stabilization [11]. A functional endothelial layer is imperative to maintain such balance and prevent thrombogenesis, as well as to control the inflammation and the phenotype expression of the cells infiltrating the vascular wall [15,20]. Here, we will review the cases that lead either to failure or to regenerative pathways.

### 5.1. CASE A: A Fibrotic Rigid Tissue Surrounds the Graft, or the Graft Is Occluded Due to a Stabilized Fibrotic Thrombus

#### 5.1.1. Fibrotic Tissue Surrounds the Graft: Fibrotic Capsule Generation—The Microstructure of the TEVG Avoids Cell Infiltration and a Dense Collagen Layer Is Formed

In a desired outcome, during the first weeks after implantation, inflammatory cells are attracted to the external layers of the TEVG from the perivascular tissue. Depending on the microstructure, the infiltration of hematopoietic stem cells (HSC), monocytes that will transform into macrophages, and other kind of leukocytes will occur to promote the generation of a de novo tunica adventitia rich in capillaries to provide the adequate influx of cells into the graft required for the vascular wall regeneration [21]. For instance, for porous and fast-degrading structures such as in saphenous vein VGs, cells within adventitial tissue have been reported to upregulate the expression of the transforming growth factor- β1 (TGF-β1), and bone morphogenetic protein 2 (BMP2), with a concomitant downregulation of metalloproteinase 1 (MMP1). Furthermore, high vascularized walls have been reported to present cells expressing the Neuron-glial antigen 2 (NG2) related to innervation, CD44 antigen related to cell attachment and the Smooth Muscle 22α (SM22α) related to the development of a contractile phenotype of smooth muscle cells (SMCs), which are all required for vascular wall regeneration [22]. However, there is a fine line between regeneration of failure of VGs, but it will likely depend on the balance of the amount and types of infiltrated cells.

In contrast, if the microstructure does not allow cell infiltration and it is resistant to biodegradation, it is likely that a fibrotic capsule will be formed. Although there is a lack of evidence of this process in TEVGs, according to the behavior of the cells in different biomaterials, we can propose different hypothesis that will need to be confirmed. In this scenario, M1 macrophages attached to the surface will increase the release of proinflammatory cytokines such as IL-4 and IL-13, and their excessive fusion will lead to foreign body giant cells (FBGCs) [23]. FBGCs adhere to vitronectin, released by platelets from their activation in the first days and adsorbed onto the inner and outer surfaces of the TEVGs. FBGCs will then release metalloproteinases (MPP 2 and MPP9) to degrade the TEVG biomaterial. Although the presence of FBGCs will always remain, high populations will have deleterious effects on the regenerative pathways of a TEVG. M1 and FBGCs will maintain the release of IL-1, IL-4 and IL-6 IL-10, which are chemoattractant for fibroblasts, and the concomitant release of TGF-β and platelet-derived growth factor (PDGF) will induce fibroblast proliferation [16].

If the biomaterial resists degradation and inhibits cellular infiltration, during the following months fibroblasts and myofibroblasts it will release collagen types I and III, as well as some proteoglycans, syndecans and the tissue inhibitor metalloproteinase (TIMP), ultimately maintaining and promoting collagen deposition; this has been reported in SV grafts in which myofibroblasts like cells express Thrombospondin 1 (TSP-1) [24]. As the cells increase the collagen production, a thick fibrous capsule is created around the vascular graft, avoiding vascularization and the numbers of cells inside the tissue decrease.

**Figure 1:**
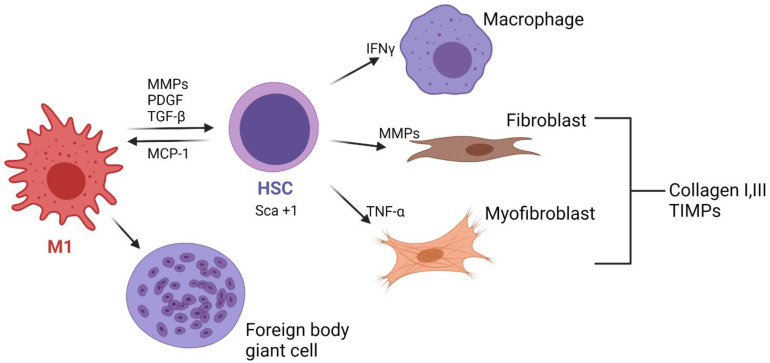
Cellular events in the generation of a fibrotic novo adventitia around the vascular grafts due to maintained M1 activity. If the TEVG exhibits resistance to degradation, M1 macrophages release pro-inflammatory cytokines that will stimulate macrophage fusion into foreign body giant cells. In another mechanism, the hematopoietic stem cells infiltrating the TEVG will differentiate towards macrophage such as cells, fibroblasts and myofibroblasts, thereby generating collagen deposition and inducing the TEVG contraction (Created with BioRender.com, accessed on 3 February 2022).

The differences between the generation of a de novo adventitia and fibrous tissue depend on the vascularization degree and the gene expression. It has been reported that Sca+1 cells (Spinocerebellar Ataxia Type 1 Protein), which are adventitial progenitors, are associated with the pathogenesis of cardiovascular diseases and have been found in the failure of SV VGs due to the fibrotic capsule generation. This gene has been related to promote the transdifferentiation of SMCs to myofibroblasts [25]. In this sense, myofibroblasts could induce a decrease in the vessel lumen, and the changes in the hemodynamic behavior will also increase the inflammatory response. Nevertheless, this hypothesis needs to be confirmed. Furthermore, it has been reported that in the maturation of arteriovenous fistulas, myofibroblast have been related to the failure of the graft as a result of the continuous release of the tumor necrosis factor (TNF-α), interferons (IFNγ) and MMPs. At the same time, if hypoxia occurs due to the damage of the vasa vasorum of the perivascular tissues, adventitial cells can be recognized by the expression of hypoxia-inducible factor 1-alpha (HIF1α)-promoting SCA+1 cell generation [26,27]. SCA+1 cells have not only reported in affecting the contractile phenotype of the SMCs but also have been shown to affect the lipid metabolism on immune cells contributing to calcium deposition and would lead to the TEVG wall calcification [27]. Figure 1 summarizes the cellular events in the generation of a fibrotic capsule around TEVGs.

##### Clinical Indications, Outcomes and Common Treatment Procedures

The formation of a fibrotic capsule surrounding an implanted device is commonly known as a response of an exacerbated foreign body reaction, and it is common in non-regenerative devices. However, for regenerative purposes the presence of a thin collagen matrix surrounding the tissue might hamper blood perfusion and nutrition of the newly populated scaffold; it will also modify the mechanical properties of the TEVG, which could cause flow disruptions [19]. Furthermore, grafts surrounded by fibrotic tissue have higher difficulty in showing a compliant response to pressure changes, and this can be observed in echography as jet-like flows through the graft, with a clear difference between the deformations from the graft in comparison with the native vessel.

The confirmation of the presence of such tissue can be achieved by histological analysis of a biopsy taken at the surrounding tissue of the implantation site. The histological analysis will show a dense layer of type 1 collagen and low cell contents. Lately, it has been suggested that fibrotic predictors could indicate the presence of IL-6, IL1β, TNFα, IL-8, IL-10 and TGF-β1 [28,29]. However, as reviewed earlier, markers such as TSP-1, SCA and HIFα might be fibrosis predictors.

**Figure 2:**
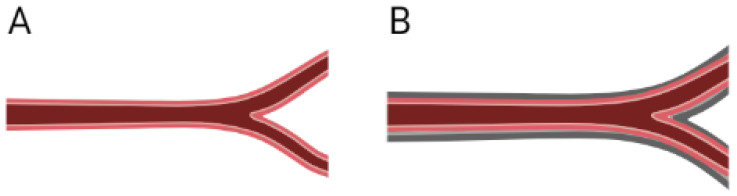
When the remodeling process of the graft induces a fibrotic novo adventitia (**A**) Physiological remodeling of the vascular wall, with thin non-fibrotic novo adventitia (**B**), the outermost external layer of the remodeling vessel is mainly composed of dense fibrotic tissue. This implies that its compliance and therefore capacity to adapt to pressure changes is impaired. Furthermore, the newly formed vessel is surrounded by a thicker, more rigid and denser layer that becomes an obstacle for cell migration from the perivascular tissue to the wall of the graft and limits oxygen and nutrient availability to the cells recently infiltrated in the graft. This not only limits the vascular wall remodeling but also the endothelial lining formation and maturation. (Created with BioRender.com, accessed on 3 February 2022).

If the presence of the fibrotic capsule is confirmed, the TEVG will fail and will need to be explanted and replaced if the flow is compromised, although there is a lack of data regarding the occurrence of this event in TEVGs due to the few clinical trials. We have found that in non-biodegradable grafts used as arteriovenous fistulas (AVF), close to the 17% have been explanted with a thick fibrous layer involving fatty acid tissue that represents up to 12% of all the causes of explantation due to the fibrous tissue infiltrating into the intima caused by the persistent punctures [30,31]. The absence of healing processes of non-biodegradable grafts that induce fibrotic deposition on the surface of the graft could be prevented by the integration of the natural materials of TEVG. However, if surface interactions promote fibrotic deposition, there is no mechanical continuity from the graft to the native vessel; TEVGs can be susceptible to fibrotic neo adventitia. Figure 2 shows a brief scheme of the possible implications of a fibrotic capsule on a TEVG.

#### 5.1.2. Fibrotic Thrombus

During the first weeks of implantation, small thrombus might be formed over the luminal surface of the TEVG; depending on whether this thrombus evolves into a stable fibrotic thrombus or not, the partial occlusion might become permanent and consequently limit the blood flow through the TEVG; as a result, hypoxia is common in the downstream regions of the TEVG. Under physiological conditions, controlled fibrinolysis takes place during the first 24 h after thrombus formation. Neutrophils release plasmin and MMP9 to degrade the thrombus, also acting as chemotactic molecules for monocytes-macrophages, inducing phagocytosis of other thrombus components. If this event does not occur, a fibrotic thrombus might be generated [32].

Although we have not found reports of fibrotic thrombous in TEVGs, fibrotic thrombus has been reported to be the main cause of non-biodegradable vascular grafts, the majority of which occur within the first and second year after implantation. Therefore, this outcome might be considered and avoided on TEVGs depending on its application [31,33]. For instance, in AVFs, ECM has been detected on the sites of puncture, suggesting that the cells from the fibrotic thrombous might inoculate through the multiple punctures [31]. Nevertheless, there are other possible factors that might increase the stability of an arterial thrombus, including the increase in the thrombin concentration, as well as a high concentration of coagulation factors such as F12a and the vWF in the bloodflow, typical in patients with acute coronary syndrome (ACS) and hyperlipidemia [34].

**Figure 3:**
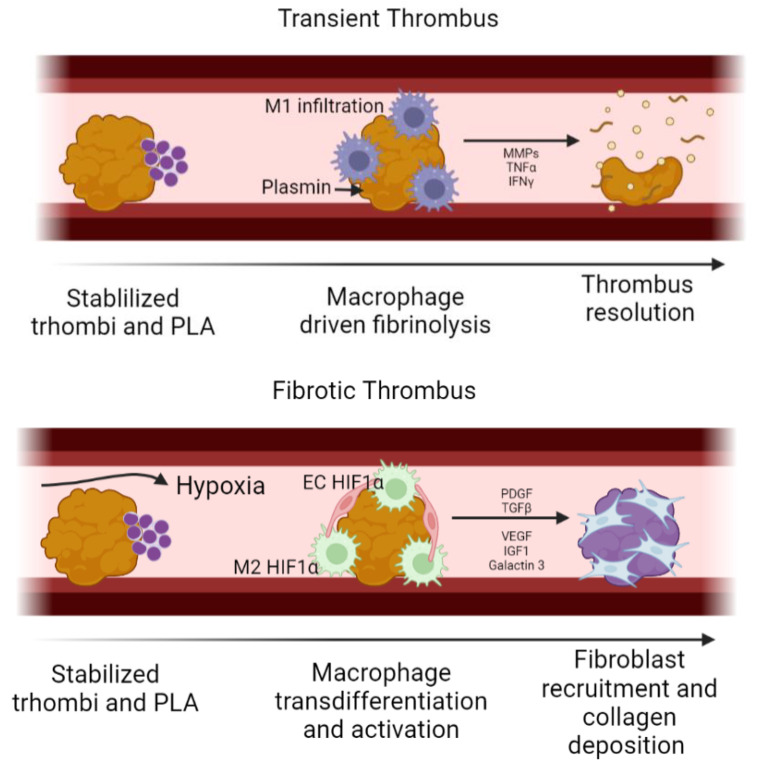
Proposed cellular events related to fibrotic thrombus generation on a TEVG. Upper image: transient thrombus is degraded by the action of M1 macrophages. However, a thrombogenic material with extended thrombus formation can promote hypoxia in the downstream cells infiltrating the vascular wall. Lower image: under hypoxia, M1 macrophages expressing the HIF1α increase the production and release of VEGF, causing angiogenesis. Hypoxia also induces M1 transdifferentation to M2 phenotype, which will release growth factors for extracellular matrix (ECM) derived fibroblast remodeling into the thrombus. (Created with BioRender.com, accessed on 3 February 2022).

However, a fibrotic thrombus in TEVGs might occur in a similar fashion as the thrombo-fibrotic remodeling on thromboembolic lesions [35]. Even if the initial thrombus is partially degraded, it is possible that the platelet leukocytes aggregates (PLA) formed during the initial thrombogenesis inhibit the complete thrombus resolution, prolonging its presence. In this case, the reduced blood flow causes a low shear stress and a hypoxic environment. Bochenek et al. [36] found that fibrotic thrombi have a high infiltration of macrophages and endothelial cells HIF1α + among macrophages and endothelial cells. An increase in HIF1α has been correlated with an increase in cell infiltration and angiogenesis inside the thrombus. This has been thought to proceed by the release of VEGF from the initial stages of inflammation. The continuous activity of HIF1α on macrophages promotes its polarization towards a M2 phenotype. In this regard, both endothelial cells and M2 macrophages maintain an interplay through P-selectin and E-selectin, allowing cell recruitment. Growth factors released from activated platelets also recruit endothelial cells and M2 macrophages, which, in turn, activate the surrounding cells, inducing fibroblast differentiation through the release of TGF-β, PDGF, vascular endothelial growth factor (VEGF), insulin-like growth factor (IGF-1) and Galactin-3 [37]. In this way, while macrophages slowly degrade the thrombi, fibroblasts deposit collagen, changing the final composition towards a fibrotic thrombus [38,39]. Therefore, the prevention of HIF1α+ cells might be key to avoid the development of a fibrotic thrombus in TEVGs. In the same way, it has been reported that the Urokinase-type Plasminogen Activator (uPA) and the Tissue Plasminogen Activator (tPA) produced by endothelial cells and monocytes are efficient in resolving thrombi in murine models, thereby avoiding fibrotic thrombus, and could be considered as potential targets [39]. Nevertheless, to our knowledge there are no studies in TEVGs reporting the development and gene expression inside a fibrotic thrombus; this would be key to understand how to improve this outcome in a long term. Figure 3 shows a schematic of the cellular events related to fibrotic thrombus generation on a TEVG.

##### Clinical Indications, Outcomes and Common Treatment Procedures 

If a fibrotic thrombus is interrupting the normal flow, it can be observed clinically [31]. Doppler images can show an increased velocity at the regions of the graft with reduced lumen area resulting from thrombus deposition. Physicians can diagnose the reduction of the flow to the distal tissue from the obstruction and indicate an intervention to dissolve or physically remove the thrombus.

**Figure 4:**
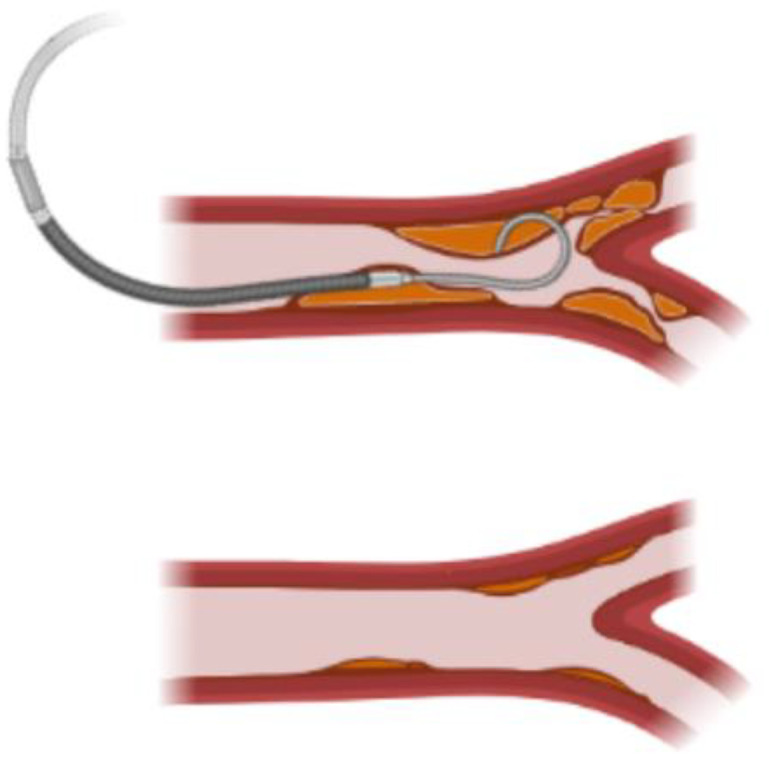
Secondary patency in TEVGs is achieved by the intervention of the graft by removing the clot with a catheter. This recuperates the lumen of the graft and allows perfusion, giving the graft a second opportunity to allow the initiation of regeneration processes without hemodynamic impairments (Created with BioRender.com, accessed on 3 February 2022).

This is the case for AVFs, in which two patency levels have been described according to the fibrotic thrombus formation. Primary patency is defined as the patency of the graft from the implantation moment to the first intervention to recover the surface area, while secondary patency is defined as the recovered area of the lumen after an intervention occurs. This intervention can be related to the removal of a thrombus plaque via catheterization. Secondary patency of thrombosed prosthetic vascular access grafts proceeds by aggressive surveillance, monitoring and endovascular management [40,41]. Figure 4 shows an scheme of the current procedure for patency recovery.

To avoid the formation of a fibrotic capsule or a fibrotic thrombus, chemical and physical modifications of the biomaterials have been proposed on different biomaterials. Chemical modifications include coatings with slow-releasing antifibrotic drugs, such as halofuginone, pirfenidodone and matisinib, inhibiting the collagen synthesis and myofibroblasts activation [42,43]. Likewise, other authors have also included the use of interference RNAs for Rapamycin and Col1 [44]. However, this should be considered carefully given that collagen deposition is also required for the regenerative responses desired for a TEVG. For instance, it has been reported that pirfenidone antifibrotic effects are related to an antioxidant activity. Thereby, antioxidant molecules can be considered instead [45].

Physical changes have also been reported; apart from providing a proper porous structure that allows cell infiltration, soft implant surfaces with elastic modules lower than 2 kPa can inhibit the profibrotic expression of the TGF-β1 induced by mechanical stimuli [46], confirming the importance of using highly compliant materials for TEVGs fabrication.

### 5.2. CASE B: Hemodynamic Conditions Are Unstable, and There Is No Sign Regenerative Induction

On preclinical models, the flow stability to maintain the graft patency is a determinant factor of the success or failure of the vascular graft. The flow regime and the resulting velocity profiles and shear stresses, as well as the cyclical circumferential deformation caused by the pulsatile flow, are positive mechanosignals in the implantation region microenvironment. It has been reported that regarding the wall shear stress (WSS), a laminar flow and a high shear stress promote a regenerative response in TEVGs, while small turbulent flows trigger low shear stress and boundary-layer detachment, impairing the physiological mechanosignaling required for a regenerative response. Dolan, et al., Abe. J.-I. et al. and Fan. L. et al. [47,48,49] suggested that vascular pathology is not only related to the binary behavior of shear stress but also to a lower and upper outrange of physiological values. Therefore, the wall shear stress gradient (WSSG) is also a relevant factor on the induction of an inflammatory or a regenerative response due to an acceleration or deceleration of the blood flow causing differential gene expression in ECs, SMCs and macrophages [47].

#### 5.2.1. Low Shear Stress Produces Turbulent Flows

Diameter or mechanical properties mismatches between the native blood vessel and the TEVG might create a turbulent or oscillatory flow. This can be also the case of complex geometries in curvatures, bifurcations, and branching points in the artery tree. Furthermore, thrombus and anastomosis regions could act as an obstacle to the blood flow, changing the velocity profiles. Under these conditions, the upstream laminar flow profile changes towards a turbulent flow with boundary layer separation.

LaMack et al. [50] reported that pro inflammatory genes associated with increased leukocyte adhesion such as intercellular adhesion molecules (ICAM), monocyte chemoattractant protein-1 (MCP1) and the activity of c-jun were upregulated in endothelial cells under low WSS and positive WSS gradients [15,47]. The unfavorable effect of low WSS on the regenerative response in TEVGs can be explained by attenuation of NO release and an increase in the reactive oxygen species (ROS) concentration, which provides a pro-inflammatory environment [12]. In contrast, while physiological shear stress maintains the production of antioxidant enzymes, low WSS has been associated with a downregulation of the proteins involved in the redox equilibrium in the cells of the vascular wall [20].

#### 5.2.2. Critically High Wall Shear Stress and Wall Shear-Stress Gradients (WSSG) Avoid Inflammation Resolution

Although these mechanisms have not been widely studied in TEVGs, it has been found that high WSS combined with positive WSSG [47,51] (i.e., accelerating flow) could promote matrix degradation by the increase in the expression of MMPs and a variety of disintegrins, such as A disintegrin and metalloproteinase with thrombospondin motifs (ADAMTS1), affecting the wall integrity and promoting aneurysm generation [47,52,53]. On the other hand, high WSS and negative WSSG (i.e., deceleration) appear to maintain the ECM integrity [14]. A positive WSSG implies a stretching force over the cells, while the negative generates a compressive force. The difference between the directions of these forces has a conformational effect on the cell mechanoreceptors [54]. For this reason, the interplay of these forces plays a central role in cell adhesion, proliferation and apoptosis, thereby determining the fate of the TEVG. For instance, mechanically stimulated ECs in TEVGs have also shown an increase in Micro RNAs 551b-5p through the early growth response-1 pathway, as well as the MicroRNA 21 in SMCs, reducing its excessive proliferation [55].

Table 1 summarizes the effect of baseline, low and critically high wall shear stress over the macrophage phenotype expression and its impact on the regenerative pathways.

#### 5.2.3. Clinical Indications, Outcomes and Common Treatment Procedures

The evaluation of the hemodynamic state can be observed through echography or angiography. The evaluation of the shear stress can be estimated by measuring the diameter of the vessel and fluid properties, and the velocity profile can be calculated using mathematical models [57].

It is possible that the altered hemodynamics are not only related by a mismatch between the TEVG and the adjacent vessel but can be also related to the presence of a fibrotic thrombus or an excessive rigidity in the anastomosis. This can be corrected with a secondary patency intervention in which hemodynamics may improve, promoting the regenerative pathway [41,47,48,49].

### 5.3. CASE C: Hemodynamic Conditions Are Stable and the VG Has Not been Rejected, but There Is No Sign of a Regeneration Process: The VG Is Inert but Stable

In this case, the prevalent conditions between the TEVG and the adjacent vessel is a laminar flow regime with a high baseline shear stress, i.e., between 10 and 20 dynes/cm^2^ (1–2 Pa). However, there is limited regeneration, even with the presence of M2 and fibroblast; meagre endothelial cells on the lumen are characteristic of this failure case. In this case, there is absence of biochemical signaling, either due to an inappropriate micro-architecture or a lack of bioactivity of the TEVG, limiting the ability of cells to begin repopulating the scaffold.

#### 5.3.1. Protein Coating but No Cell Proliferation

If the TEVG has bioactive properties but there is no cell proliferation, this failure might be caused by undesired protein adsorption. Depending on the type, the protein coating on the surface of VG regulates the interaction between cells and the biomaterial surface, promoting or inhibiting cell adhesion. This coating occurs in early stages after the TEVG implantation due to their affinity for specific moieties on the surface and their surfactant behavior. For instance, hydrophobic materials such as polyurethanes (PU) easily attract and bind host proteins from the plasma and the interstitial fluid. Surfactant nature of proteins emerges as an effect of their large size, wide array side chain functional groups and partial unfolding [58]. These properties allow noncovalent interactions between the proteins and the biomaterial surface that can last for the first weeks, avoiding cell adhesion [59,60].

The predominant proteins that adsorb to biomaterials, especially to polymers, are albumin, immunoglobulin G and fibrinogen [61]. If fibrinogen or albumin are mainly absorbed, they can create a thin layer, blocking cell adhesion. Furthermore, the maintained adsorption of fibrinogen over almost any biomaterial surface will promote platelet and monocyte/macrophage adhesion, favoring the proinflammatory state [58].

#### 5.3.2. Microstructure Arrangement Avoids Cellular Infiltration

A common finding in pre-clinical animal models is limited regeneration; although this can be related to the limits of the study times or the failure before completion, it can be also due to the TEVG inability to be biocompatible, either by its mechanical properties, macro- or microstructure, or lack of bioactivity. According to our previous results, the tissue regeneration of small intestine submucosa (SIS)-based TEVGs on a swine preclinical model revealed a strong dependency on the manufacture and implantation parameters. In one of our contributions, we demonstrated that cell repopulation depends on preservation or removal of the stratum compactum layer of the intestine, and the dehydrated or hydrated state of the graft [62]. This study showed that preserved and dehydrated (PD) scaffolds had 100% patency at 3 months. However, the grafts also demonstrated limited regeneration signals. PD grafts showed non-occlusive thrombi, had mild inflammatory reaction and kept their tubular structure. However, these grafts had the lowest rates for regeneration measured in terms of low vascularization, scarce macrophage (CD206+ for M2 and CCR7+ for M1), fibroblast infiltration and absence of endothelial cells. Additionally, PD scaffolds were mechanically stiff and fragile despite the preservation of tubular structure. Even though the PD graft remained patient, this case can be considered as a failure due to the lack of regenerative response due to the microstructure failing regarding allowing cellular infiltration. In these cases, one of the currently studied alternatives for TEVGS is the implantation of cell-seeded tissue scaffolds to be populated with adult progenitor or induced pluripotent stem cells from the patient. However, although they have shown efficiently overcoming the first phase compared with unseeded grafts, their performance is not different during the maturation period of the TEVG, probably due to a microstructure incapable of sustaining cell infiltration or proliferation for vascular wall regeneration [63,64].

In both cases, the role of the acute inflammatory response in its ability to recruit endothelial cells and stem cells that will begin the vascular wall remodeling is highlighted. Without a proper initial acute inflammation, the TEVG will not sustain regeneration [11]. For instance, scaffolds with higher pores allow for better cell infiltration, and the immune response can be controlled avoiding chronic inflammation, to identify if the chronic inflammation is avoided and the macrophage-driven degradation is taking place in a controlled mechanism different markers can be analyzed. In an animal model or an in-vitro model, controlled inflammation has an increase of M2 markers, however, identifying markers for M1, and overexpression of the NADPH oxidase 2 (NOX 2) can be indicative of the reactive oxygen species production in immune cells related to a chronic inflammatory response [22,65]. Furthermore, foreign body giant cells (FBGCs) can also be identified on the surface of biomaterials without a pro-regenerative microstructure. To detect these type of cells, newly specific markers can be recognized, such as the Human Leukocyte Antigen—DR isotype (HLA-DR), CD98 and CD86 [66].

Still, different approaches have been proposed to induce the regeneration of small-diameter TEVGs avoiding chronic inflammation showing enhanced regeneration of the different cell populations in the vascular wall. The use of organoselenium compounds’ [67] functionalization with cytokines for M2 such as IL4 [67] and resolving D1 [68] antioxidants [69,70] and even small extracellular vesicles with micro RNAs 126 and 145 related to the VEGF function, all with the concomitant suppression of M1 [71], has been reported.

#### 5.3.3. Clinical Indications, Outcomes and Common Procedures

The absence of a regenerative process will impair the possibility of the graft to actively respond to the hemodynamic stimuli and to remodel the vascular wall.

**Figure 5:**
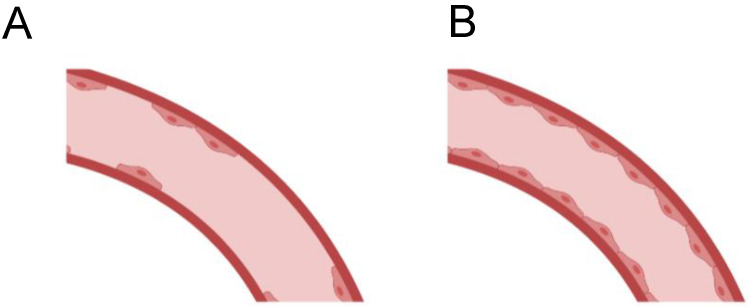
(**A**) If there is a homogeneous deposition of endothelial cells in the inner surface of the graft, this will mature into a functional endothelium. (**B**) However, it is possible that only isolated proendothelial cells get attached to the inner surface of the graft. These cells will not be able to form endothelial lining and will end up being detached from the wall without modulating vascular wall remodeling (Created with BioRender.com, accessed on 3 February 2022).

If the reason for the absence of reendothelialization is not related to flow and the TEVG has bioactive properties, antioxidative therapy and immunologic stabilizers could be proposed as suitable treatment to promote regeneration when the flow stimuli is stable [72]. New trends in regenerative medicine include systemic support for regeneration including diet, rest, and controlled healing (Figure 5).

### 5.4. CASE D: Hemodynamic Conditions Are Stable, and the Graft Shows Markers for Regeneration—There Is a Repopulation Form Endothelial Cells

The vascular wall remodeling, which is the main indicator of regeneration and integration of the TEVG, depends on the formation and the maturation of a functional endothelium. Therefore, the first indicator for a successful TEVG is its capacity to sustain a reendothelialization process of the inner surface of the graft as well as angiogenesis in the vascular wall.

#### 5.4.1. Endothelialization

Endothelialization, which is the formation of a lining of endothelial cells over the surface of a TEVG, is a process that is still under study. Thus far, our knowledge of the process indicates that progenitor endothelial cells (ECs) in humans can arrive from either the anastomotic sites (transanastomotic origin) or the granulation tissue surrounding the TEVG (transmural origin). Transanatomotic ECs’ migration is limited to a few millimeters from the anastomosis site towards the center of the graft, but the ECs coming from the surrounding tissue are responsible for inducing capillary generation along the vascular wall. In consequence, the endothelialization of the TEVG lumen mainly depends on the deposition of progenitor ECs from the blood flow [73,74].

##### Endothelial Precursor Cells (EPCs) from the Blood Stream

There is still a knowledge gap regarding the origin of the EPCs in TEVGs. Some authors have suggested that circulating EPCs are derived from a common HSC precursor in the bone marrow. These kind of EPCs are recognized by the expression of CD34, CD31, CD133, VEGFR2 and eNOS. Other markers include their microvesicles with adhesion molecules and receptors such as ICAM-1, VLA-4, CD44, CD154, L-sectin, VEGFR2, CD34, CXCR4, CD117 and CD135 [75]. In general, EPCs have been reported to perform anti-apoptotic, proliferative and pro-angiogenic effect over other EPCs through signaling pathways such as eNOS and MAPK [75].

Alternatively, other authors have hypothesized that origin of ECPs is mesenchymal stem cells (MSCs). MSCs have been isolated from bone marrow and have been demonstrated to have endothelial differentiation capacity when grown under endothelial conditions. These cells are positive for VEGFR2, vWF and VE-Cadherin. MSC-derived ECs have been studied in tumor development, and it is likely that their transdifferentiation occurs mainly through paracrine mechanisms [76].

Lately, a separate myeloid origin for EPCs has been suggested. Although myeloid cells express CD14 and ECs fail to do so, it has been shown that when grown under endothelial conditions, the expression of CD14 in monocytes decreases with a concomitant increase of endothelial cell markers such as vWF and NOS. Smith et al. [77] demonstrated reendothelialization of an arterial VG by circulating monocytes. Therein, the authors suggested that one type of M2 macrophage, called M2 endothelial cell or M2e, can turn into an EC when activated by some particular signals, such as VEGF. In this case, the M2e would maintain both the CD14 and endothelial markers (CD144 and VEGFR2) [78]. Kutikhin et al. [75] reported similar findings in which human peripheral blood cells are able to differentiate towards endothelial-colony-forming cells with similarities to mature vascular endothelial cells. This was demonstrated by transcriptomics analysis of monocytes and HUVECs [79].

The EPCs’ migration and adhesion is also dependent on WSS and WSSG; under shear stress, it is mediated specifically by integrins β1 and β3 and by the PI3K/Akt/mTOR pathway. After an initial decrease of fiber stress due to shear stress, integrin β1 pathway increases the stress in fibers to promote cell alignment in the direction of the flow [80]. TNF-α and hypoxia were found to initiate the adhesion process of EPCs to the intima layer due to the affinity of VLA-4 to VCAM-1 [75]. The reendothelialization ability of EPCs mediated by shear stress has also been correlated to the regulation pathways of Tie2/PI3K/Akt [75]. Other identified EPCs migration mechanisms are via Ang-2/Tie2/PI3K/Akt/eNOS [75].

The attachment of EPCs to the TEVG surface has been reported to be a function of fluid viscosity and density, as well as the concentration of cells in blood and the surface properties of the device. Laminar flow promotes EPCs’ adhesion under physiologically-induced shear stress. In TEVGs, higher endothelialization capacity and reduced IH tendency were found under laminar flow but not under static conditions for EPC cultures [80]. The TEVG topography also plays a significant role in the reendothelialization process as it has been demonstrated through micro and nanostructures as modulators of adhesion, but more importantly in the maturation and proliferation of ECs [81].

**Figure 6:**
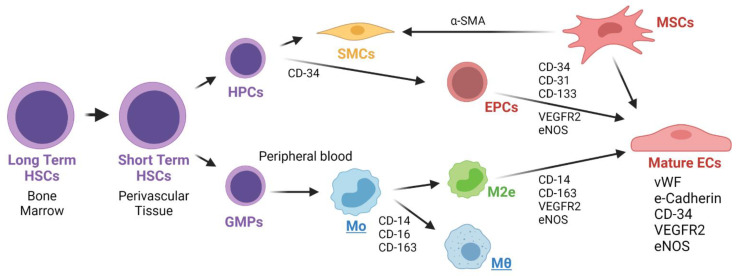
Different proposed origins for Endothelial Progenitor Cells (EPCs). From hematopoietic stem cells (HSC) to hematopoietic progenitor cells (HPCs) that can then differentiate to SMCs and EPCs, HSCs can also differentiate towards granulocyte monocyte progenitors (GMPs), in which monocytes differentiate to monocytes (Mo) and then to an M2e macrophage expressing endothelial markers. Mature ECs can also be derived from mesenchymal stem cells (MSCs). (Created with BioRender.com, accessed on 3 February 2022).

In this regard, anisotropic topographies such as grooves contribute to the elongation and alignment of cells, therefore promoting native conformation of the endothelial lining [82]. Furthermore, the relative direction of the anisotropic features with respect to the blood flow will also affect the preferential migration and source of progenitor endothelial cells. If this alignment is perpendicular, migration from the edge of the graft is favored over the flow migration, while if for parallel arrangement the dependency on flow conditions in higher [81]. Figure 6 shows the different proposed origins for Endothelial Progenitor Cells (EPCs) that might contribute in the TEVG endothelialization.

##### ECs Initial Differentiation

The differentiation and maturation of EPCs into ECs an important sign of endothelialization and regeneration. Some of the markers of mature ECs are the enhanced expression of CD31, vWF and Tie2, as well as the reduced expression of CD133 and CD3 [70]. In addition, the activation of pathways such as PI3K, Akt, mTOR, ERK1/2, JNK and p38/MAPK has been reported to be linked with the capacity of EPCs to form pro-regenerative tube-like structures when under shear-stress influence. Additionally, maturation has been linked with the expression of proteins related to EC adhesion, such as VCAM-1/CD106, ICAM-1/CD54 and E-selectin/CD62E [75]. Fast endothelialization and the generation of a SMCs layer on vascular grafts seeded with EPCs previously exposed to shear stress have been reported in a sheep carotid artery replacement model [75].

Shear stress levels could trigger anti-thrombotic and anti-atherosclerotic capacities of the endothelium. Under a shear stress of 25 dynes/cm^2^ (2.5 Pa), ECPs elevate the expression of COX-2, PGI2, tPA, KLF2, CDD141, eNOS and the release of NO, while there is a decrease in the expression of thromboplastin, endothelin-1 and IL-8 [75]. The EPCs proliferation has been mainly correlated with the expression of vascular endothelial growth factor receptor 2 (VEGFR2), CD34 and CD133. The mature ECs that form the endothelium have been reported to express VEGFR2, CD31 and vWF markers. The mature ECs resulting from peripheral blood circulating EPCs express VEGFR2, CD34 and CD133 markers. Hematopoietic stem cells from the bone marrow and non-hematopoietic stem cells in the blood vessels, liver and spleen originate and are the precursors of peripheral circulating EPCs [75]. Table 2 summarizes the markers for EPCs and mature ECs.

To overcome the acute inflammatory responses and start a physiological regeneration process of the vascular wall, the TEVG microenvironment for endothelialization can be optimized. This means that the structure of the TEVG needs to be tailored for cell adhesion and migration through the balance of inflammatory cues that promote progenitor endothelial cells towards a functional endothelium. Moreover, the hemodynamic conditions at the implantation site should promote expression of mature endothelial phenotypes.

##### Endothelial Lining Formation

The maturation of the endothelial lining is highly dependent on flow conditions. If the TEVG shows a matching geometry and elasticity with the native vessel, and the flow conditions are physiological, the newly formed endothelial lining will be likely to express a healthy phenotype that will be ultimately responsible for regulating vascular wall remodeling.

The directionality of pulsatile flow has been reported to cause the alignment of actin, tubulin and intermediate filaments in ECs, which have been linked with their functionality [83]. In this case, the laminar flow exposed in Phase II case B is accomplished during a time span of at least the first month to promote endothelialization and vascular wall regeneration.

The turnover on gene expression from EPCs to mature ECs is a desired marker in TEVG regeneration process upon implantation [75]. In addition to the growth factors and cytokines from the inflammatory response (VEGF, IGFB, Endoglin, Semaphorins, etc.), shear stress stimuli have been found to induce differentiation of EPCs into mature ECs. The shear stress-induced mechanosignaling occurs due to cytoskeletal rearrangements of EPCs under moderate and high WSS ranges [75]. For example, bovine aortic ECs (BAECs) subjected to a WSS of 35 dyn/cm^2^ in vitro showed orientation along their longitudinal axes and parallel to the flow direction. However, when exposed to WSS of 284 dyn/cm^2^, non-evident alignment was identified after 24 h and 36 h [47]. Other authors reported reduction of EC alignment when exposed to WSS below 130 dyn/cm^2^ Pa [77], and reduction of cell density under high WSS (around 300 dyn/cm^2^) with statistically significant difference with respect to low WSS (around 40 dyn/cm^2^). On the contrary, EC proliferation was increased under high WSS [47].

However, WSS ranges for optimal alignment and monolayer formation under in vitro conditions differ significantly from the physiological reported values. Even WSS values as high as 300 dyn/cm^2^ have shown optimal morphological outcomes on ECs cultures [84]. However, some authors report that the exposure of ECs to moderate WSS, as promoted by laminar flow under physiological conditions in straight arterial segments, provides stimulation of their function by increasing the expression of factors such as the KLF2 (Kruppel-like factor 2). This factor induces the expression of endothelial NO synthase (eNOS) and inhibits expression of pro-inflammatory molecules such as VCAM-1 and E-selectin. In addition, the KLF-2 has been reported as anti-thrombotic under laminar flow conditions [85].

On the other hand, turbulent geometries develop low WSS due to non-homogenous velocity profiles close to the VG wall. Laminar and unidirectional flows present a WSS of around 15 dyn/cm^2^ (1.5 Pa), while disturbed flow produces a WSS of around 5 dyn/cm^2^ [7,86]. The low WSS and turbulent flow not only under-stimulate but also detrimentally change the morphology and function of ECs. Round-shaped ECs expose the vasculature, accelerate apoptosis and promote a pro-inflammatory response. The detrimental effect of positive WSSG combined with high WSS on ECs was also [87] reported for animal models in regions near to bifurcations and aneurysms, where geometrical parameters provide turbulent flow regimes [84]. Table 3 summarizes the combined effect of WSSG and WSS on cell survival. 

The maturation of the endothelium is therefore dependent on the dynamic mechano-signaling provided by the pulsatile flow. In vitro studies have shown that the acceleration of flow also affects the proliferation, viability, and alignment of ECs [84]. For example, by combining low WSS and high WSSG in endothelial cells, it has been shown that it is possible to achieve increased migration, proliferation and activation of transcription factors compared to static cultures [84]. Other reports demonstrated that high wall-shear stress and cyclic shear stress increase the expression of Angiopoietin-2 and its receptor Tie2, which leads to activation of the PI3K/Akt pathway. This results in the expression of eNOS and the subsequent increase in the released NO from ECs [87,88].

#### 5.4.2. Clinical Indications, Outcomes, Common Procedures and Design Markers That Need to Be Considered in Translational Studies

The success in the endothelialization process can be observed clinically by the absence of thrombus formation and a compliant vessel that shows continuity in the pulsatile wave from the graft to the native tissue, as observed in echography.

## 6. Phase III: The Vascular Graft Behavior within Operation—Vascular Wall Remodeling and Device Maturation

If the vascular graft has initiated the endothelialization process, the inflammatory response is stabilized, and if the TEVG is patent, its success is evaluated regarding to its ability to support the vascular wall remodeling. This will ensure that the mechanical properties of the graft will allow compliance as a response to blood flow while avoiding calcification or loss of elasticity.

During this stage, the causes of failure are therefore related to the balance of the remodeling process of the vascular wall. One complication is the uncontrolled proliferation of smooth muscle cells, which results in IH and ultimately in the loss of patency. If endothelium formation is not continuous and the intimal layer is covered by patches of cells, the ability to control blood flow is impaired, which may lead to hemodynamic and homeostasis issues. Furthermore, if the inflammatory interactions of the vascular wall continue to be present, there might be calcium deposition along the lumen, resulting in an increase in rigidity and leakage.

### 6.1. CASE A: The Vascular Wall Remodeling Turned to Excessive Smooth Muscle Proliferation and the Graft Is at Risk of Occlusion Due to Intimal Hyperplasia (IH)

The impaired flow conditions presented in Phase II, Case B continue to occur in a manner involving positive feedback, promoting turbulent flows; low shear stresses; and, consequently, boundary layer detachment. Therefore, VG patency loss is found mainly due to the excessive proliferation of the synthetic phenotype of SMCs, and eventually the vascular graft fails. There is also a mismatch between native vessels and the vascular graft in terms of stiffness and diameter adaptability [5,48,89]. Moreover, there is an absence of appropriate physiological mechano-signaling, which results in alterations of EC proliferation and eNOS expression. WSS ranges as well as WSSG are important markers of mechano-signaling to promote or hinder matrix degradation, and cell proliferation and maturation [4,84].

Low WSS vales have been reported for complex geometries such as curvatures, bifurcations and branching points in the artery tree, as well as under pathological conditions of multidirectional, turbulent and oscillatory blood flow [49,80]. The formation of an endothelial lining depends on the evolution of the inflammatory process; nevertheless, the general paracrine and endocrine signals that promote the transformation of macrophages into a functional type are systemic, while the loss of patency due to excessive muscle proliferation and arteriosclerosis are mainly localized phenomena. This means that SMCs proliferation and formation of plaques must respond to changes in the local hemodynamic conditions. Contrary to pulsatile shear stress, which is typical in laminar flow in straight vascular regions, the presence of oscillatory shear stress in branches and bifurcations has been identified for regions of endothelial dysfunction [90]. Furthermore, the magnitudes of pulsatile flow found physiologically are more than 10 times higher than those for oscillatory stress situations.

When the geometry of the VG and the hemodynamic conditions at each end of it cause blood flow separation from the vascular wall (boundary layer separation), the shear stress over the endothelial lining is dramatically reduced, thereby leading to a lack of ECs stimulation. As a compensatory mechanism, the vascular wall will increase its thickness by an excessive proliferation of smooth muscle cells and ultimately the loss of lumen patency. This is, for example, the case of VGs for arterio-venous fistulas, in which the high pressure on the arterial side leads to lowering velocities, and therefore to reduced shear stimulation to the endothelium [91].

Intimal hyperplasia (IH) is one of the causes of late failure of vascular grafts and can be attributed to continued lack of stimulation (low shear stress) [40]. IH occurs when there is a disproportionate proliferation and invasion of smooth muscle cells (SMCs), which causes an excessive contraction that ultimately closes the vessel’s lumen. The overexpression of MMP-9 and MMP-2 has been implicated in the development of IH and occurs within the first to 4 weeks of implantation. This is given that MMPs are chemoattractants of SMCs and promote invasion of the VGs. Turner et al. selectively suppressed the gene expression of MMP-2 and MMP-9 in an in vitro model [92]. Results showed that SMCs from saphenous vein decreased their migration; however, individual inhibition failed to fully recover the SMCs migration, thereby suggesting that both are synergistically operating to induce SMCs invasion. In more recent works, pharmacological therapies with the inhibitors of MMP-9, simvastatin and doxycycline demonstrated a decrease in migration and invasion of alpha-smooth muscle actin (α-SMA)-positive cells (contractile phenotype of SMCs) in vivo [93,94]. Conversely, the overexpression of TIMP 2 and 3 has also been reported in the development of IH on TEVGs; although TIMPs will inhibit the activity of MMPs, they promote cell proliferation. The balance between MMP2s and TIMPS should be better studied on TEVGs to understand and prevent the IH outcome (X). However, IH has a multifactorial pathophysiology that increases with ROS, and the activation of mechanoreceptors on the surface of ECs.

According to our own experience, IH is a very common cause of failure for small diameter vascular grafts (<5 mm). Biological scaffolds patency is highly influenced by manufacturing variables such as decellularization media and time, as well as synthesis protocols. Extracellular matrix (ECM) vascular grafts such as those made of small intestinal submucosa (SIS) have been reported to present varying patency rates between 0 and 100% in accordance with luminal surface modifications and hydration state [95]. One modification of the luminal surface that has reported to induce a significant increase in patency (from 0–13% to 88–100%) is maintaining the stratum compactum layer of the intestine (dense collagen layer) [96]. However, the presence of the highly rich collagen layer produces poor cellular infiltration, which restricts the regeneration of tissues. Hydration of ECMs scaffolds is another important factor to promote cellular adhesion; however, dehydrated SIS sheets have also found application as bioinks, vascular grafts, hemostatic plugs, and base materials to produce regenerative medicine scaffolds [76,97]. The dehydration contributes to the preservation of the material, reducing risk of contamination and degradation of components over time; in addition cellular adhesion must be controlled to avoid adverse outcomes such as IH [98].

#### 6.1.1. Anastomosis Integration and Mismatch on Mechanical Properties

**Figure 7:**
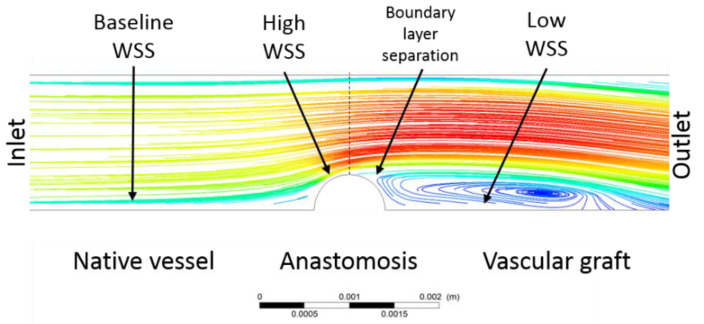
Schematic of the regions for baseline, high and low wall shear stresses at an anastomotic site. The boundary layer separation site is also shown. The anastomosis represents an obstacle that perturbs the laminar flow conditions downstream of its location. Created with ANSYS (2016). ANSYS Fluent-CFD Software|ANSYS, accessed on 3 February 2022).

Stiffness and diameter adaptability differences between native vessels and vascular grafts can lead to alterations in the WSS and circumferential stress during operation [86]. The anastomosis angle can be involved in the generation of flow disturbances and vortices that decrease the WSS, affecting the hemodynamics and cell response [38]. Therefore, mismatches in the compliance lead to alterations in radial and circumferential stresses that eventually resulted in the failure of VGs [39]. Additionally, the anastomotic sites are critical regions where diameter interferences result in substantial flow profile changes. Preclinical animal models (e.g., rats) have reported that anastomosis and other surgery factors are critical for implant success and particularly the patency outcome [39,47]. CFD analysis have suggested that, depending on the geometry and flow parameters, flow obstacles induced by anastomosis might lead to high shear stress values upstream and low ones downstream [49]. The low ones are a direct consequence of turbulent flow caused by the obstacle. Figure 7 shows a CFD schematic of shear stress values along an anastomotic site. The high shear stress values are usually greater than those found in straight arteries (i.e., >3 Pa) [49].

#### 6.1.2. Clinical Implications, Outcomes and Treatment

The excessive SMC proliferation will result in a reduction of the lumen, which in echography will appear as an increase in the magnitudes of the velocity through the graft. If there is a simultaneous hemodynamic alteration because of thrombus formation of rigid anastomosis, if solved, the body will regulate the SMCs proliferation by itself and the wall thickness will be reduced.

### 6.2. CASE B: The Chronic Inflammatory Response Promotes Calcification—Similar to Atherosclerosis, the Vascular Graft Is Completely Occluded and Rigid and Fails to Comply with Its Functions

Laminar and unidirectional flow results in shear stress values of around 15 dynes/cm^2^, while disturbed flow produced leads to values of around 5 dynes/cm^2^. High WSS values have been correlated with an atheroprotective function, mainly due to the maintenance of homeostasis in the endothelia and the appropriate alignment and signaling of ECs. In contrast, low WSS values are considered atheroprone (i.e., lower than 1 Pa), according to previous reports [80], because turbulent flow induces morphological and functional changes in ECs. In this regard, round EC morphologies have been shown to expose the vasculature accelerate apoptosis and promote a proinflammatory response. The pathological response of the impaired complex flow is, therefore, endothelial injury and atherosclerosis.

#### Atherosclerosis

Almost all the synthetic vascular grafts exhibit atherosclerosis over time, as evidenced by the presence of calcifications. Atherosclerosis in vascular grafts occurs as in native vessels, whereas calcified plaque is deposited over the inner surface of the vascular graft. This process leads to a significant occlusion of the vessel lumen. Calcified plaque formation usually occurs under chronic inflammation conditions in which the monocytes circulating in blood infiltrate the inner layers of the vessels and the matured macrophages will transform into foam cells [15]. Macrophages control the lipoprotein and cholesterol levels in blood through a complex mechanism in which a signaling pathway allows the lipids to transform and secrete as cholesterol. However, under chronic inflammation, the low-density lipoproteins in blood oxidize (ox-LDL) and remain stored in pro-inflammatory type I macrophages to generate the foam cells [16]. Excessively proliferated SMCs migrate towards the lumen of the vessel, and the VG can transform them into foam cells. These cells are thought to be responsible for causing calcium deposition, hardening, and occlusion of the vessel lumen as they release vesicles that promote mineral nucleation once in contact with the material [16].

**Figure 8:**
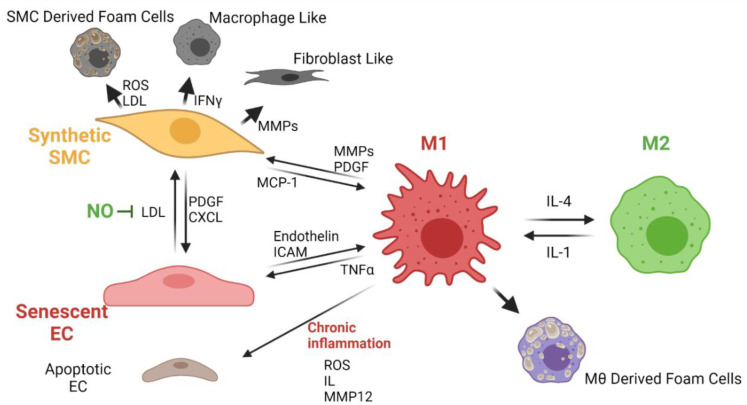
Chronic inflammation and atherosclerosis. If there is resistance of the biomaterial to degradation, the oxidative stress produced during inflammation induces changes in the M1 calcium and lipid metabolism. This causes an increase in the intracellular calcium accumulation. Chronic inflammation also induces EC senescence, which is characterized by a decrease in the NO release and an activation of the LDL metabolism. An increase in LDL in addition to the activity of MMPS and PDGF induces a synthetic phenotype of SMCs. This promotes transdifferentiation into SMC derived foam cells, macrophage-like cells or fibroblast-like cells (Created with BioRender.com, accessed on 3 February 2022).

Foam cell generation in vein grafts was observed within the first year of implantation. After that time, the mineral nucleation avoided perfusion of nutrients to cells on the vascular graft, which resulted in the development of an intraplaque necrotic area between 2 to 5 years after the surgery. In some situations, angiogenesis processes might occur within the atherosclerotic plaque, causing destabilization and rupture, possibly resulting in hemorrhage [17]. Figure 8 shows the proposed cellular interplay related to chronic inflammation and atherosclerosis development in TEVGs.

### 6.3. CASE C: The Graft Is Yet to Be Fully Occluded, the Previously Initiated Process Has Stopped and Only Some Regions of the Graft Are Populated by Endothelial Cells; the Vascular Wall Lacks a Physiological Structure, and Although Patency Has Been Reduced, Oxygen Delivery Is Not Impaired—There Is an Imbalance between the Different Cells Populating the Vascular Wall

The remodeling process of the vascular wall could result in one of two outcomes. Either there is no repopulation of cells in the graft or the repopulation of the vascular graft is unbalanced. In the first case, the material resides at the implantation site as a non-regenerative graft with low chances of success. In the second case, the excessive SMCs proliferation leads to the progressive detachment of adhered endothelial cells.

Some studies that report a patchy endothelium formation on the lumen usually explain this phenomenon by the presence of microvessels going from the external environment into the lumen. This microcirculation is caused by the angiogenic response of inflammatory processes within the porosity of the graft, which serves for cell infiltration but occurs heterogeneously [18]. Furthermore, migration of ECs can be impaired by several CDVs where proliferation capacities might be severely limited. In some pathologies, senescent ECs remain on a G0 phase of the cell cycle, which means that they exhibit limited proliferation and their expression profile changes [33]. In this regard, the nitric oxide synthase (NOS) expression decrease is accompanied by an increase of endothelin 1 (ET-1) expression. While NOS promotes vasodilatation, ET-1 promotes vasoconstriction. This state can be generated by inflammation, oxidative stress, trombogenic events and turbulent blood flow, which can be typically found when a vascular graft is implanted [35].

#### Critically High Wall Shear Stress and Wall Shear-Stress Gradients in Endothelial Cells and Smooth Muscle Cells (WSSG)

Understanding the implications of wall shear stresses over the endothelium formation and the degradation of the matrix is critical for vascular graft design since, for example, WSS values higher than 50 dynes/cm^2^ are common in arteries connected to arteriovenous fistulae, in the throats of stenosis, and in collateral arteries. WSS values of around 300 dynes/cm^2^ are typically found in bifurcations and curvatures of native vessels. The highest turnover of ECs under high WSS occurs near apices of bifurcations as a result [84] of expansive vascular remodeling in the vasculature. While high WSS values promote EC proliferation [85], the same regime has been reported to increase apoptosis and an to induce an injurious effect over cell integrity. Similar findings have been also reported for WSS values below the physiological baseline [63]. Table 4 summarizes the effect of previously categorized ranges of WSS over ECs, SMCs and macrophages.

### 6.4. CASE D: The Remodeling of the Vascular Wall Is happening under Physiological Velocities, and There Are Signs That a Complete Remodeling of the Vascular Wall and Integration Is Achievable

Thus far, the most successful graft is the autologous one. Commonly, the harvested vessels are veins, but they often replace arteries and, consequently, even under ideal integration conditions, need maturation to compensate for the arterial operation.

A recent comparison between the only commercially available TEVG, which is based on bovine carotid artery, showed no significant patency differences with respect to the autologous graft [99]. Evidence of other tissue-engineered vascular grafts in long-term patency has been evaluated in pre-clinical models without success. The success of a TEVG is measured in terms of several indicators for remodeling, including operation under reduced pressure gradients over maturation time, absorption of the original material, structural organization of muscle fibers and deposition of extracellular matrix, which provide compliance along with expression of functional cell phenotypes [100].

Flow conditions are also important determinants of TEGVs performance as they define the evolution of the endothelial lining during the regeneration process. High success rates have been associated with stable TEVGs working under pulsatile flow, where atheroprotective mechanisms are prevalent. Atheroprotection is defined as the capability for inhibition of inflammation while accelerating proliferation and migration of ECs [90].

In this regard, an appropriate hemodynamic state would promote the expression of pro-endothelial factors. For instance, the stimulation of the endothelial lining under physiological pulsatile stress has led to the activation of endothelial nitric oxide synthase (eNOS). Furthermore, under these conditions, protective mechanisms against atherosclerosis start taking place, including upregulation of cholesterol biosynthesis through a lipid metabolism pathway mediated by the SREBPI protein [90]. Additionally, under pulsatile flow, proliferation of endothelial cells is stimulated.

#### Endothelium-Mediated Homeostasis

**Figure 9:**
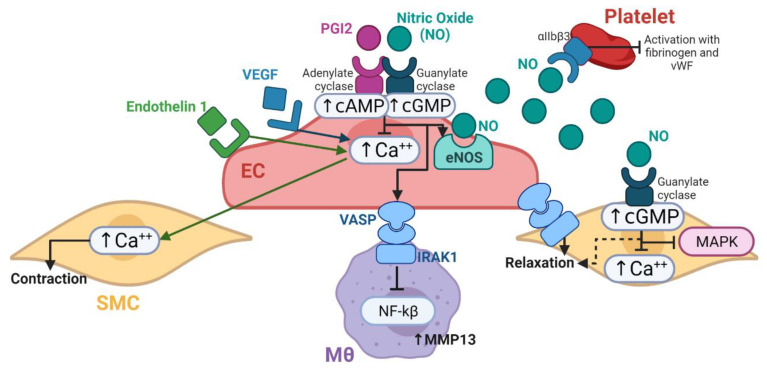
Endothelial cells’ regulatory activity in the vascular wall. Different stimuli induce the synthesis and release of nitric oxide (NO), which regulates the cells involved in the regeneration process of the vascular wall. Paracrine responses: 1. NO inhibits platelet activation when in contact with fibrinogen or vWF. 2. NO inhibits proliferatory pathways in SMCs and induces muscle relaxation. Autocrine response: NO induces the synthesis of the vasodilator-stimulated phosphoprotein (VASP). 1. VASP induces muscle relaxation. 2. VASP inhibits NFKβ activation in macrophages. Endothelial cells modulate thrombogenesis, muscle tone and inflammation (Created with BioRender.com, accessed on 3 February 2022).

Healthy endothelial cells are capable of adjusting to the vessel diameter by providing compliance through the release of NO and prostacyclin (PGI2). NO has a paracrine and autocrine activity. First, NO and PGI2 interact with platelet receptors and increase the concentration of the cyclic guanosine monophosphate (cGMP) and cyclic adenosine monophosphate (cAMP), thereby inhibiting signaling and mobilization of calcium, which is required for the platelet activation and auto- amplification. Second, on SMCs, NO leads to an increase in cGMP, which allows relaxation and inhibits MEK and ERK kinases responsible for SMCs over-proliferation. The autocrine activity of NO is the release of proteins such as the vasodilator-stimulated phosphoprotein (VASP), which also induces SMCs relaxation. Recent reports have suggested that VASP can interact with the interleukin 1 receptor (IRAK1) in macrophages, thereby leading to inhibition of NFκβ mediated proinflammatory pathways. The early inflammatory events are required to initiate angiogenesis and therefore formation of endothelial lining. Once this endothelium is formed, the vessel starts remodeling in response to pressure-flow stimulation. Next, the progenitor cells in the area will form the outer layers of the vessel. The challenge of regenerative VGs is to retain this endothelium formation over time and to sustain contractile smooth muscle cells in the tunica media. Figure 9 shows the proposed cellular interplay related to the patency maintenance in TEVGs by the means of endothelial cells action in platelets, smooth muscle cells and macrophages.

## 7. Conclusions

While synthetic grafts are expected to allow blood perfusion without active response to complex hemodynamic conditions, and their failure is related to occlusion or periimplanation complications such as infections, regenerative vascular grafts or TEVGs fail to fully comply with the required functions to overcome the lack of physiological integration of the graft with the adjacent the native vessels. Therefore, the success of the regenerative process to create a neo-vascularized wall is only possible when the excessive inflammatory response is overcome while maintaining blood perfusion, and a signaling microenvironment of the implant is optimized to trigger the regenerative metabolic pathways mediated by the inter cell phenotype crosstalk.

Regenerative vascular grafts can only begin to remodel the vascular wall if a proper reendothelialization process is taking place. This depends on a relatively low oxidative stress environment and an imparted wall shear stress stimuli to improve the mechanotraduction sensed by all the cells infiltrating the vascular wall, as well as the modulation of the inflammatory responses mediated by a macrophage/mononcyte switching to pro-regenerative phenotypes capable of participating in angiogenesis processes.

Due to the difficulties in diagnosing the cause of the vascular graft failure on in-vivo and clinical conditions, here we propose to improve the preclinical testing of the TEVGS to determine the development of functional cell phenotypes during the neo-vascualr remodeling at the TEVGs through the analysis of the cell interactome with their surroundings, assisted by trending technologies as genomics, transcriptomics, proteomics and metabolomics, to fabricate next generation of biomolecular-cues-guided TEVGs.

**Figure 10:**
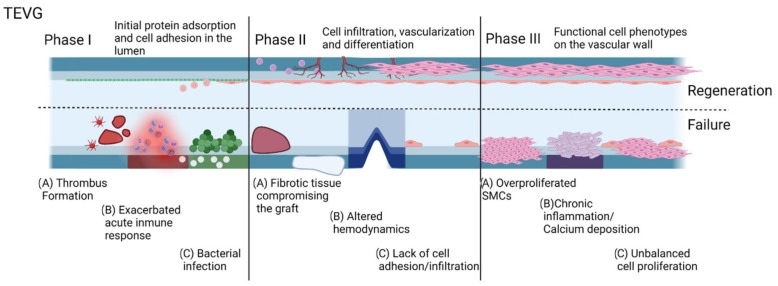
Macroscopic implication of causes of failure. Phase 1: the inflammatory and peri-implantation conditions have been stabilized; there is an initial protein adsorption and cell adhesion in the lumen. The causes of failure might be (A) thrombotic events, (B) an exacerbated acute immune response and (C) bacterial infection. Phase 2: the graft has overcome the first phase, and the hemodynamic conditions are homogeneous. The causes of failure include the (A) fibrotic tissue generation in the thrombus or in the periphery of vascular grafts, (B) altered hemodynamic changing the cell phenotype expression, and (C) the lack of cell adhesion or infiltration avoiding the vascular wall repopulation. During this phase, the chronic inflammation responses are led by fibroblasts and foreign body giant cells to encapsulate the biomaterial. Phase 3: Once the endothelial lining covering the inner surface is established, the cell and remodeling of the vascular wall will occur. The causes of failure might be due to (A) the excessive SMC proliferation (hyperplasia) by a compensation mechanism of the hemodynamic conditions, (B) calcification of the graft from a chronic inflammatory response, and (C) an unbalanced cell proliferation due to a lack of bioactive signals. After 2 to 5 years, the lack of balance on the regenerative signals and the cell responses will maintain the chronic inflammation state and the failure of the vascular graft will occur. For all the cases, the development of a functional endothelial phenotype will promote proinfllammatory pathways while at the same time that enhancing regeneration. The current challenge is to balance the different cell responses to improve endothelialization (Created with BioRender.com, accessed on 3 February 2022).

The role of aTEVG is to compensate the microenvironment deficiencies to rationally guide cell behavior, including phenotype switching and proliferation rates, while the regeneration processes should be enhanced by the modulation of the microstructure followed by topography and bulk mechanical properties tailored to allow cell infiltration and maturation as well as well as provide compliance, the inclusion of cell adhesion surfaces with compensating antioxidative–anti-inflammatory signals, and thrombus formation impairment coatings. Figure 10 shows a brief summary of the macroscopic events leading to either TEVG regeneration or TEVG failure. 

## Figures and Tables

**Table 1:** Effect of baseline, low and critically high shear stress on macrophage and inflammation resolution according to the literature [47,51,56].

		Macrophages	
	Value [Pa]	Downregulated Genes	Upregulated Genes
Physiological limits	1.5–2.4 (Straight arterial segments)	Leukocyte	M2 phenotype
Low shear stress	0.1–1	M2 phenotype	Leukocyte M1 Macrophages and neutrophils NF-Jb Ets-1, IL-1 MCP-1 MMP-2 and -9 Selectin-mediated leukocyte rolling
Critically high shear stress	3–34		M1 Macrophages and neutrophils Selectin-mediated leukocyte rolling, platelet activation, adhesion and aggregation Genes for fibrinolysis proliferation, and matrix remodeling

**Table 2:** Different markers for EPC and mature ECs on TEVGs.

EPC Common Markers	Mature EC Markers
MMP-1 MMP-9 VEGFR2+/CD34+ VEGFR2+/CD133+ CD34+/CD133+	VEGFR2 VEGFR1 PECAM1 vWF Tie2 EphB2 Hey1/2 ALK1 COX-2 CD31 VE-Cadherin

**Table 3:** Outcome of the combination of WSSG and WSS over endothelial lining based on a rabbit animal model of a bilateral common carotid ligation, and in vitro and CFD approaches [47,51,56].

	Positive WSSG (0 to 600 Pa/mm)	Negative WSSG (−600 to 0 Pa/mm)
WSS higher to 50 Pa	Associated with cell apoptosis and aneurism damage	Associated with cell apoptosis
WSS lower to 50 Pa	Associated with cell apoptosis	Associated with cell apoptosis

**Table 4:** Effect of baseline, low and critically high shear stress over EC, SMCs and macrophages.

		ECs		SMCs	
	Value [Pa]	Downregulated genes	Up-regulated genes	Downregulated genes	Up-regulated genes
Physiological limits	1.5–2.4 (Straight arterial segments)	TNF-α mediated JNK E-selectin TXNIP PKC	KLF-2 Prostacyclin ERK1/2 PECAM-1 SHP2 Glutathione Grx P38 ERK5 PGI_2_ Tpa CD31 CD144 eNOS vWF	Cell cycle inhibitor p21 BMP4 Smad1 Smad3 p53	Pro-inflammatory cytokines cyclin D1 Akt pathway IL-11 Smad7
Low shear stress	0.1–1	eNOS p47phox subunit of NADPH oxidase	(Inflammatory markers): VCAM-1 ICAM-1 E-selectin Endothelian-1 MCP-1 PDGF (Platelet derived)	α-SMA SM22 SM-MHC Smoothelin calponin	Proliferation alpha SM-actin
Critically high shear stress	3–34		eNOS PDGF FGF-2 (MMPs)-2 and -9 plasmin-induced metallothionein dismutases ADAMTS1, ADAMTS6) and serine proteases (tPA and uPA) SAC inhibitor Ki-67	SMC Apoptosis	FGF-2 mRNA (MMPs)-2 and -9

## Data Availability

Not applicable.
